# Supplementary figures and images for: Genome-wide analysis of lectin receptor-like kinases family from potato (Solanum tuberosum L.)
Source: PeerJ. 2020 Jun 10;8:e9310. doi: 10.7717/peerj.9310 (PMC7293193; doi:10.7717/peerj.9310)

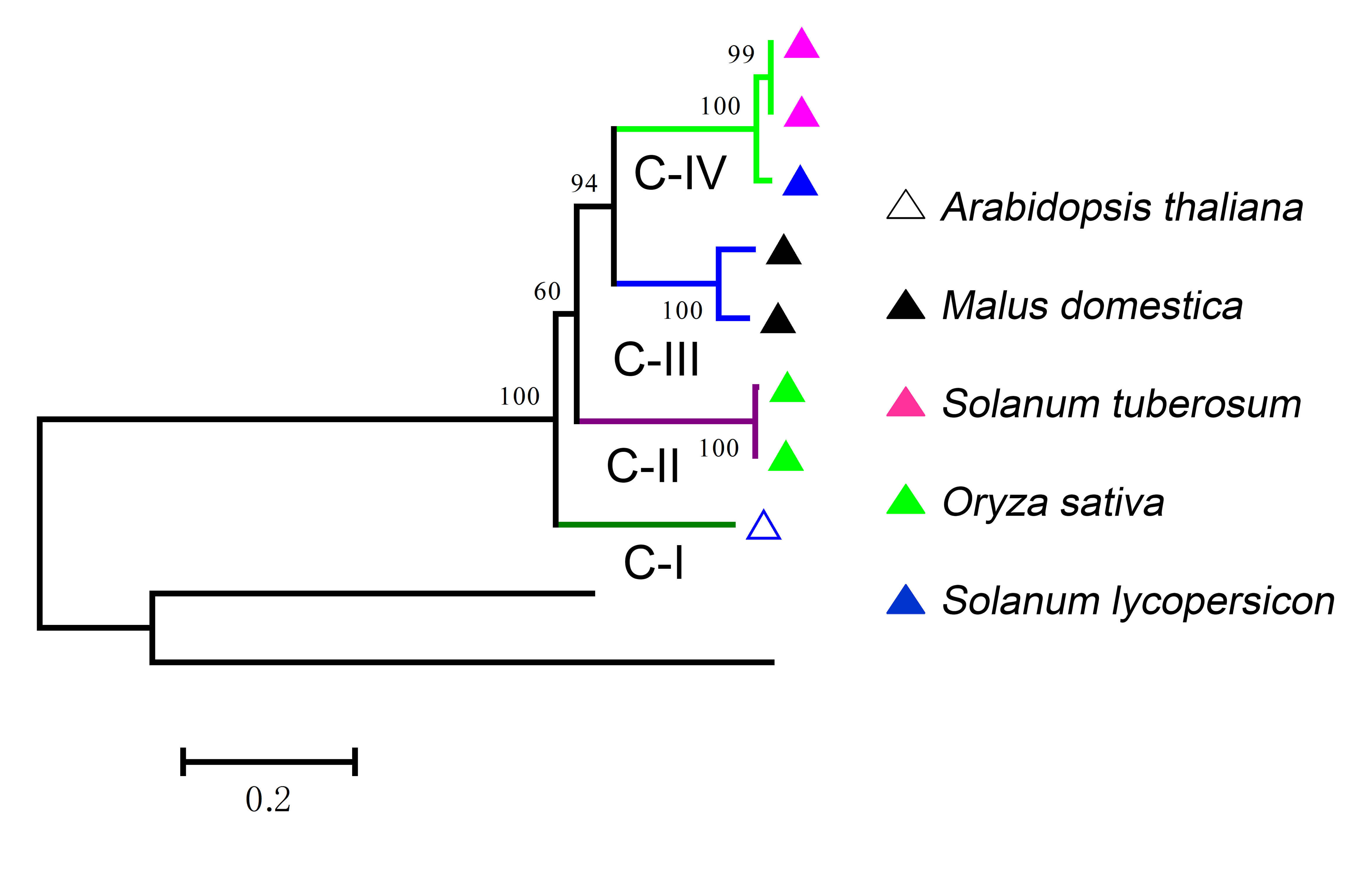

Supplement: Supplemental Information 1 — Different color was used to distinguish different subgroups. The neighbor-joining (NJ) method was used to analyze the evolutionary trees. [file peerj-08-9310-s001.png]
